# Steroid hormone regulation of EMP2 expression and localization in the endometrium

**DOI:** 10.1186/1477-7827-6-15

**Published:** 2008-04-09

**Authors:** Madhuri Wadehra, Monica Mainigi, Shawn A Morales, Rajiv G Rao, Lynn K Gordon, Carmen J Williams, Jonathan Braun

**Affiliations:** 1 Department of Pathology and Laboratory Medicine, David Geffen School of Medicine at UCLA, Los Angeles, California 90095, USA; 2 Center for Research on Reproduction and Women's Health and Department of Obstetrics and Gynecology, University of Pennsylvania, Philadelphia, PA 19104, USA; 3 Department of Ophthalmology, David Geffen School of Medicine at UCLA, Los Angeles, California 90095, USA; 4 Molecular Biology Institute, David Geffen School of Medicine at UCLA, Los Angeles, California 90095, USA

## Abstract

**Background:**

The tetraspan protein epithelial membrane protein-2 (EMP2), which mediates surface display of diverse proteins, is required for endometrial competence in blastocyst implantation, and is uniquely correlated with poor survival from endometrial adenocarcinoma tumors. Because EMP2 is differentially expressed in the various stages of the murine and human estrous cycle, we tested the hypothesis that the steroid hormones progesterone and estrogen influence EMP2 expression and localization.

**Methods:**

Frozen human proliferative and secretory endometrium were collected and analyzed for EMP2 expression using SDS-PAGE/Western blot analysis. The response of EMP2 to progesterone and estradiol was determined using a combination of real-time PCR, SDS-PAGE/Western blot analysis, and confocal immunofluorescence in the human endometrial carcinoma cell line RL95-2. To confirm the in vitro results, ovariectomized mice were treated with progesterone or estradiol, and EMP2 expression was analyzed using immunohistochemistry.

**Results:**

Within normal human endometrium, EMP2 expression is upregulated in the secretory phase relative to the proliferative phase. To understand the role of steroid hormones on EMP2 expression, we utilized RL95-2 cells, which express both estrogen and progesterone receptors. In RL95-2 cells, both estradiol and progesterone induced EMP2 mRNA expression, but only progesterone induced EMP2 protein expression. To compare steroid hormone regulation of EMP2 between humans and mice, we analyzed EMP2 expression in ovarectomized mice. Similar to results observed in humans, progesterone upregulated endometrial EMP2 expression and induced EMP2 translocation to the plasma membrane. Estradiol did not promote translocation to the cell surface, but moderately induced EMP2 expression in cytoplasmic compartments in vivo.

**Conclusion:**

These findings suggest that targeting of EMP2 to specific locations under the influence of these steroid hormones may be important for integrating the molecular responses required for implantation competence.

## Background

Epithelial membrane protein-2 (EMP2) is a tetraspan protein that plays an important role in the endometrium. EMP2 is necessary for blastocyst implantation in mice [1], and aberrant EMP2 expression correlated with poor survival in endometrial adenocarcinoma patients [2]. Functionally, EMP2 has been shown to play a role in trafficking of molecules, which include integrins, major histocompatibility complex (MHC) class I molecules, and glycosylphosphatidyl inositol (GPI)-anchored proteins, to non-caveolar glycolipid-enriched lipid rafts [3-5]. These plasma membrane microdomains are important in the formation of receptor-protein complexes required for efficient signal transduction. Through this mechanism, EMP2 plays a role in the regulation of cell adhesion and invasion [3,6,7].

EMP2 is spatially and temporally regulated in the endometrium. In particular, in the mouse, a prominent increase in EMP2 expression and translocation to the apical plasma membrane occurred during the window of implantation, when physiologic effects of estradiol and progesterone on the endometrium have reached a peak [1]. Similarly, in the native human endometrium EMP2 expression is minimally detectable during the proliferative phase, but is much more highly expressed during the secretory phase [1,2]

Successful implantation of the blastocyst-stage embryo into the maternal endometrium requires a precise series of coordinated events [8]. In the endometrium, a number of specific molecules such as integrins, osteopontin, and the progesterone receptor have been implicated in implantation [9], and many of these molecules require estrogen or progesterone for activation. Endometrial epithelial cells undergo profound changes during the different phases of the estrous/menstrual cycles in response to alterations in circulating levels of estrogen and progesterone. A rising estrogen level initiates endometrial proliferation and regulates estrogen target genes in a cell-specific manner [10]. Progesterone, on the other hand, transforms the proliferative endometrium into secretory endometrium and regulates the expression of genes important for establishing endometrial receptivity and the progressive phases of implantation [11]. As the identities of the molecules important in this transition emerge, a challenge is to define the mechanisms through which they integrate and organize these biochemical changes to facilitate blastocyst attachment and implantation.

The present investigation was carried out to test directly whether or not the steroid hormones estradiol and progesterone can account for the temporal and spatial patterns of EMP2 expression. Using protein immunoblot quantitation, we confirm that EMP2 protein is elevated in primary human secretory vs. proliferative endometrium. In the human endometrial cell line RL95-2, EMP2 protein levels were induced by progesterone alone, a process inhibitable by progesterone antagonist, and augmented in combination with estradiol. Similarly, in ovariectomized mice progesterone specifically increased EMP2 protein expression and surface translocation, whereas estradiol had a minimal effect on EMP2 expression and did not induce surface translocaiton. Thus, steroid hormones induce deployment of EMP2 in both humans and mice in a manner consistent with its apparent role as an organizer of molecular components required for blastocyst implantation.

## Methods

### Human tissue

Frozen endometrial tissue samples, from proliferative or secretory phases, were obtained from the UCLA Department of Pathology archival biospecimen repository under Institutional Review Board (IRB) approval. Three proliferative (subphase unspecified) and three secretory (one post-ovulatory day 3, one late, one unspecified) phase tissue samples were obtained, homogeneized, and resuspended in Laemmli buffer. Samples were analyzed for EMP2 expression by SDS-PAGE as outlined below.

### Cell line and hormone treatment in vitro

RL95-2 (ATCC, Manassas, VA), a well-differentiated endometrial adenocarcinoma cell line, was cultured in a 1:1 mixture of Dulbecco's modified Eagle's medium and Ham's F12 medium supplemented with 10 mM HEPES, 2.0 g/L sodium bicarbonate, 0.005 mg/ml insulin, 10% fetal calf serum (Hyclone Laboratories, Logan, Utah), 2 mM L-glutamine, 1 mM sodium pyruvate, 100 U/ml penicillin, and 100 U/ml streptomycin (all from Invitrogen Life Technologies). For hormone treatment, RL95-2 cells at 60–70% confluence were washed in PBS, and subsequently incubated with steroid hormones in treatment medium (DMEM/Ham's F-12 supplemented with 0.5% charcoal-stripped FBS [Omega Scientific, Tarzana, CA], 1% penicillin-streptomycin, and 1% L-glutamine). Progesterone and 17β-estradiol (Sigma Corp., St. Louis, MO) were prepared in 1000-fold concentrated stocks in absolute ethanol, and added to treatment medium to yield a final ethanol concentration of 0.1%. In some experiments, cells were incubated with progesterone in combination with a progesterone receptor antagonist (mifepristone (RU 486); Sigma). The treatment times and drug concentrations for each experiment are indicated in the text and/or figure legends.

### Quantitative RT-PCR

Quantitative real-time RT-PCR was performed on the isolated RNA by using the Quantitect probe RT-PCR kit (QIAGEN, Valencia, CA) on the DNA Engine Opticon Monitor 2 (MJ Research Inc, South San Francisco, CA). For EMP2 genomic RNA detection, the long terminal repeat (LTR) forward primer (5'-TCCTCTCCACCATTCTCT-3'), LTR reverse primer (5'-AAACCTCTCTCCCTGCTTCA-3'), and the fluorogenic probe (6-carboxyfluorescein [6FAM]-5'-TTCTTCATCTTCGTGCT-3'-tetramethyl carboxyrhodamine [TAMRA]) were utilized for the RT-PCR. The quantity of EMP2 was calculated by interpolation from a standard curve generated by running in parallel serial dilutions of known quantities of full length EMP2 cDNA incorporated into the EGFP-N3 vector. The EMP2 signals were normalized against the housekeeping gene GAPDH using the Qiagen GAPDH Assay on Demand which included both primers and probe. The β-actin copy number was also calculated by interpolation from a standard curve generated from serial dilutions of a plasmid containing GAPDH cDNA (clone3869809; Open Biosystems, Huntsville, AL)

### Antibodies

Rabbit polyclonal antibodies generated against human or mouse EMP2 (hEMP2 or mEMP2, respectively) have been described previously [7,12]. Commercial antibodies used included estrogen receptor (ER) and progesterone receptor (PR) antibodies (all from Santa Cruz Biotechnology, Santa Cruz, CA), γ-adaptin antibodies (BD Biosciences, San Diego, CA) and horseradish peroxidase-conjugated secondary antibodies (1:1500; BD Biosciences; Southern Biotechnology Associates, Birmingham, AL). Normal rabbit sera were used as negative controls for the primary antibody.

### Immunoblot analysis

Cells were washed in PBS, collected, and resuspended in Laemmli buffer. To detect EMP2 expression, cell extracts were treated with peptide N-glycosidase F (New England Biolabs, Beverly, MA) to deglycosylate the proteins as previously described [7]. The lysates were separated on a 12% SDS-PAGE gel, and proteins were transferred to nitrocellulose (Bio-Rad Laboratories, Hercules, CA). EMP2 was detected using primary hEMP2 (1:2000) antisera followed by a horseradish peroxidase-conjugated goat anti-rabbit IgG and ECL detection reagents (Amersham-Pharmacia, Piscataway, NJ).

### Confocal microscopy

RL95-2 cells were plated onto glass coverslips (Fisher Scientific, Pittsburgh, PA) overnight. Cells were washed in PBS and then incubated for 48 hours with 25 μM progesterone, 20 μM estradiol, or a vehicle control in the treatment media described above. Cells were fixed and permeabilized in methanol at -20°C for 30 min and rehydrated in PBS. Cells were blocked in 1% normal goat serum for 45 min and incubated overnight at 4°C with the EMP2 rabbit sera (1:250) and an anti-γ-adaptin antibody (1:200) in a humidified chamber. Cells were rinsed with PBS + 0.01% Triton X-100, then incubated (2–4 hours. at RT) with fluorescein isothiocyanate (FITC)-conjugated goat anti-rabbit IgG (1:50) or Rhodamine-conjugated donkey anti-mouse IgG (1:500; Jackson ImmunoResearch Laboratories, West Grove, PA). Negative controls included incubation of cells with secondary antibody alone. Cells were copiously washed in PBS + 0.01% Triton, rinsed briefly with double deionized H_2_0, and mounted in a 3.5% n-propyl gallate-glycerol solution.

Confocal pictures were taken using a Zeiss LSM 510 confocal microscope at 600× magnification. The colocalization coefficient between EMP2 and γ-adaptin was determined using Zeiss LSM 5 PASCAL software (Carl Zeiss MicroImaging GmbH, Germany), and values represent the relative number of colocalizing pixels of γ-adaptin compared to the total number of pixels above threshold. The mean colocalization coefficient was averaged from at least 3 independent images.

### Animals and hormone treatment *in vivo*

All animals used in this study were maintained in accordance with the National Academy of Science *Guide for the Care and Use of Laboratory Animals*, with a controlled light schedule (14L:10D) and controlled temperature range. Ovariectomized female CF-1 mice (Harlan Sprague-Dawley, Indianapolis, Indiana) were treated with daily injections of 100 ng per mouse of 17β estradiol or 1 mg per mouse of progesterone dissolved in 100 μL of sesame oil, or sesame oil alone (Sigma) as previously described [13]. Mice were euthanized at various time points (indicated in text or legends), and the uteri were processed for immunohistochemistry using mEMP2 antisera or control rabbit serum. Tissue blocks were immunostained as outlined below.

### Immunohistochemistry

Uterine tissue was stained as previously described [1]. Briefly, uterine tissue was fixed in 10% formalin, embedded in paraffin, and sectioned at 5 μm thickness. Antigen retrieval was performed using 0.1 M citrate, pH 6.0, at 95°C for 20 min. The slides were incubated with mEMP2 antisera (1:250) or control rabbit serum at the same dilution overnight. The antibody signal was visualized using the Vectastain ABC kit (Vector Labs, Burlingame, CA) according to the manufacturer's instructions. EMP2 expression was detected using diaminobenzidine. Nuclei were counterstained using hemotoxylin.

### Statistics

Differences in EMP2 expression levels between test and vehicle control groups were evaluated using an unpaired student's t test at a 95% confidence level (GraphPad Prism version 3.0; GraphPad Software, San Diego, CA). To compare multiple groups, ANOVA analysis was performed using GraphPad Prism software.

## Results

### EMP2 expression in human tissue

**Figure 1:**
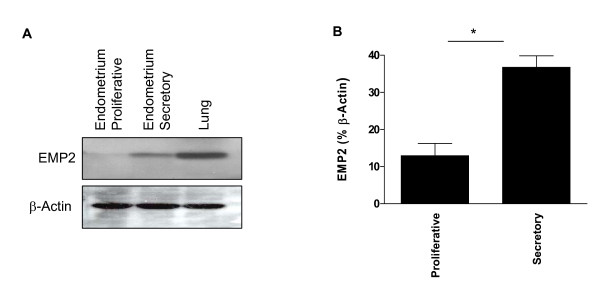
(A) Normal human endometrium specimens from subjects in proliferative and secretory phases were immunoblotted using EMP2 antisera. β-actin was used as a loading control. (B) Statistical analysis of EMP2 expression levels in proliferative (n = 3: subphase unspecified) and secretory (n = 3: one post-ovulatory day 3, one late, one unspecified) endometrial specimens from six independent patients. Data were analyzed using a 2-tailed, unpaired student t-test with a 95% confidence interval. *Changes in expression between the proliferative and secretory endometrium were significant, p = 0.038.

EMP2 expression varies both temporally and spatially during the murine estrous and human menstrual cycles, suggesting that it is regulated by the steroid hormones progesterone and estradiol. To determine the expression of EMP2 in the human endometrium, tissues were obtained from normal patients in either the proliferative (n = 3, subphase unspecified) or secretory (n = 3; one post-ovulatory day 3, one late, one unspecified) phase of the estrous cycle. Expression in the lung was provided as a control, as it is the site of highest EMP2 expression [7]. As shown in Fig. 1, EMP2 expression was strikingly upregulated (3-fold) in secretory versus proliferative endometrium (p = 0.038).

### Effect of steroid hormones on EMP2 mRNA and protein levels in vitro

**Figure 2:**
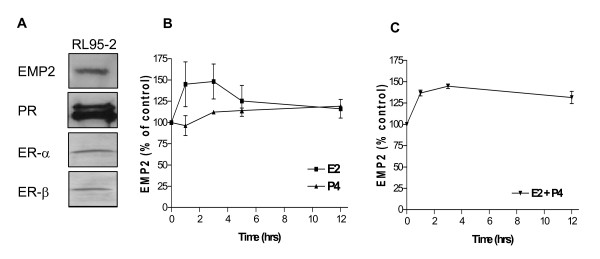
Steroid hormones induce EMP2 mRNA expression in RL95-2 cells. (A) Validation of hormone receptor status of RL95-2 cells. Cell extracts were processed by SDS-PAGE and Western blot for detection of EMP2, ER, and PR protein expression. (B and C) Temporal effect of hormone treatment in RL95-2 cells on EMP2 mRNA. RL95-2 cells were cultured with 20 μM estradiol and/or 25 μM progesterone. After 1–12 h, extracts from hormone-treated cells were obtained, mRNA was isolated, and EMP2 transcript levels were quantitated by quantitative RT-PCR. Results are expressed as the percentage increase of EMP2 over the vehicle control, and represent the average of 3 independent experiments. Error bars are SEM; p values were calculated by ANOVA. (B) EMP2 mRNA expression in response to progesterone or estradiol are alone. (C) EMP2 mRNA expression in response to a combination of estradiol and progesterone.

To assess the regulation of EMP2 expression, the well-differentiated endometrial adenocarcinoma cell line RL95-2 was utilized. RL95-2 cells express EMP2 [1] and steroid hormone receptors [14,15], and these results were validated by SDS-PAGE/Western blot analysis (Figure 2A). In order to elucidate the regulation of EMP2 by steroid hormones, estradiol, progesterone, or a vehicle control added to RL95-2 cells for 1–12 hours. Cells were harvested at different times, and analyzed using quantitative RT-PCR. As seen in Figure 2B, estradiol or progesterone alone modestly increased EMP2 mRNA, to levels that were non-significant or minimally significant by ANOVA (p = 0.3 and 0.02, respectively). The combination of estradiol and progesterone produced a similarly modest increase, but one which was more statistically significant (p = 0.002; Figure 2C).

**Figure 3:**
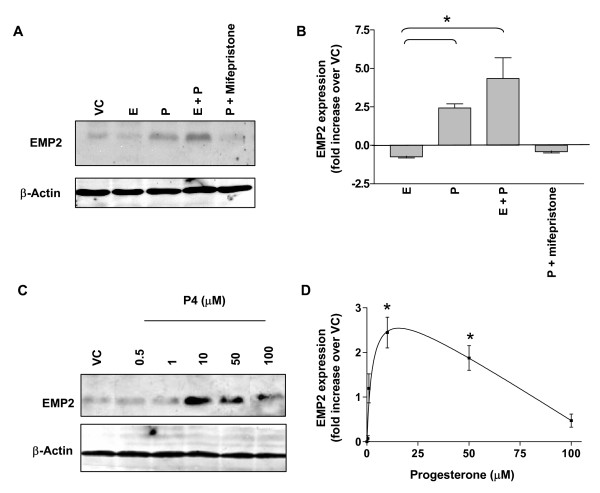
Progesterone induces EMP2 expression in RL95-2 cells. (A) Western immunoblots for EMP2 from extracts of RL95-2 cells cultured for 72 h with 25 μM progesterone (P), 20 μM estradiol (E), P plus E, P plus 100 nM mifepristone, or a vehicle control (VC; ethanol). Western immunoblot for β-actin was used as a loading control. (B) Western immunoblots from hormone-treated RL95-2 cells were quantitated for EMP2, normalized for β-actin, and data from three or more experiments were tabulated for mean and SEM. *Changes in EMP2 expression between progesterone treatment and the vehicle control were significant, p = 0.03. (C) RL95-2 cells were cultured with the indicated concentrations of progesterone (P) for 72 h, and EMP2 and β-actin expression were detected by Western immunoblots. (D) Quantitative analysis of the EMP2 response to varying concentrations of progesterone, performed as described in (B). *Changes in EMP2 expression were significant compared to the vehicle control, p < 0.05. All experiments were repeated at least 3 times with similar results.

To characterize the effects of progesterone and estradiol on EMP2 with respect to protein expression, steroid hormones were added individually or in combination to RL95-2 cells for 72 h. EMP2 expression was significantly induced by progesterone (2.5-fold compared to vehicle control), and the receptor selectivity of this response was confirmed by suppression of this response in the presence of mifespristone (RU 486), a progesterone receptor antagonist (Fig. 3A,B). In contrast to progesterone, estradiol alone slightly reduced EMP2 expression compared to the vehicle control. Combining estradiol with progesterone numerically augmented the increased in EMP2 protein observed with progesterone alone (4-fold compared to vehicle control). This response was not taken to be synergistic, since there was no significant difference between progesterone treatment alone and a combination of progesterone and estrogen treatment (p = 0.30). These findings suggest that progesterone largely accounts for the increase in EMP2 expression observed during the secretory phase of the cycle in mice and in humans [1,2]. In view of the much smaller mRNA changes, these protein findings suggest that EMP2 regulation by progesterone may be indirect (presumably by primarily targeting other cellular processes affecting post-translational fate of EMP2).

To further quantitate the effects of progesterone on EMP2 protein expression, RL95-2 cells were exposed to different concentrations of progesterone ranging from 0.5–100 μM for 72 h. EMP2 expression was selectively induced with progesterone at concentrations between 10 and 50 μM (Fig. 3C,D). Notably, progesterone at a 100 μM concentration induced ~50% cell death as indicated by trypan blue staining (data not shown). This toxicity probably accounted for the reduced EMP2 induction at this hormone concentration (Fig. 3C,D).

### Progesterone increases the surface expression of EMP2 in vitro

**Figure 4:**
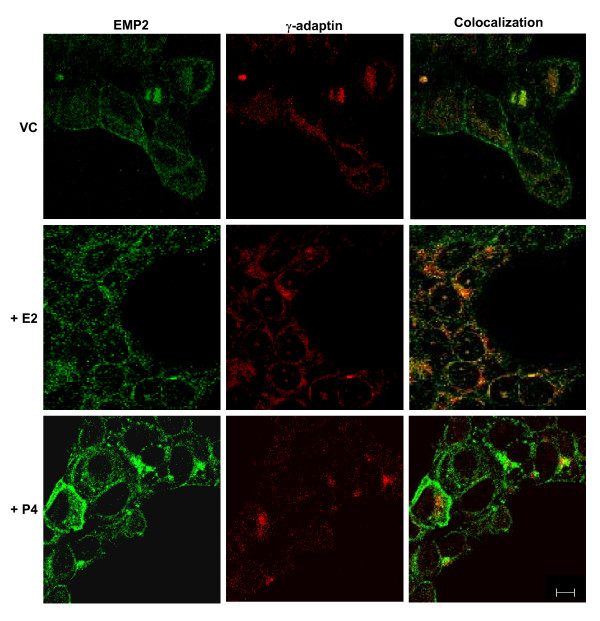
Progesterone induces surface expression of EMP2 in RL95-2 cells. RL95-2 cells were treated for 72 hours with 25 μM progesterone, 20 μM estradiol, or a vehicle control, and processed for confocal microscopy with antibodies for EMP2 (FITC) and γ-adaptin (Rhodamine). Images were captured at a magnification of 600×. Scale bar, 10 μM. The experiment was repeated at least 3 times with similar results.

To determine the localization of EMP2 protein following hormone treatment, RL95-2 cells were stained and analyzed by confocal immunofluorescence (Fig. 4). Cells were cultured in media containing charcoal stripped FBS and a vehicle control, progesterone, or estradiol for 24 hours. Cells were then stained for both EMP2 and the Golgi apparatus marker, γ-adaptin, as previous studies had suggested that EMP2 resides in this intracellular compartment [4].

The results of this analysis are shown in Figure 4. In vehicle control cells, the majority of EMP2 appeared to reside in intracellular compartments, with 31.7 ± 2% of total EMP2 colocalizing within the Golgi apparatus. As previously described, some EMP2 localization was also detectable on the plasma membrane [1]. Following estradiol treatment, less EMP2 was observed on the plasma membrane, with most EMP2 detectable in intracellular compartments, including increased colocalization in the Golgi apparatus (42.5 ± 4% of total EMP2) (Fig. 4). In contrast, following progesterone treatment, expression of EMP2 was observed predominantly on the plasma membrane. Quantitatively, this is reflected by the reduced localization of EMP2 in the Golgi apparatus (28.0% ± 0.4 of total EMP2).

### Steroid hormone effects on EMP2 expression and surface expression in native mouse endometrium in vivo

To extend these observations to native endometrium, we determined the effects of estradiol and progesterone on EMP2 expression in the uteri of ovariectomized mice. Three to five weeks after ovariectomy, mice were injected daily for up to 5 days (days 0–4) with estradiol, progesterone, or sesame oil, the vehicle used for dissolving the steroid hormones. Uteri were harvested on days 1, 2, 4, and 7 after initiating treatment (note that day 7 was 3 days following the last hormone injection) and then stained for EMP2 expression.

**Figure 5:**
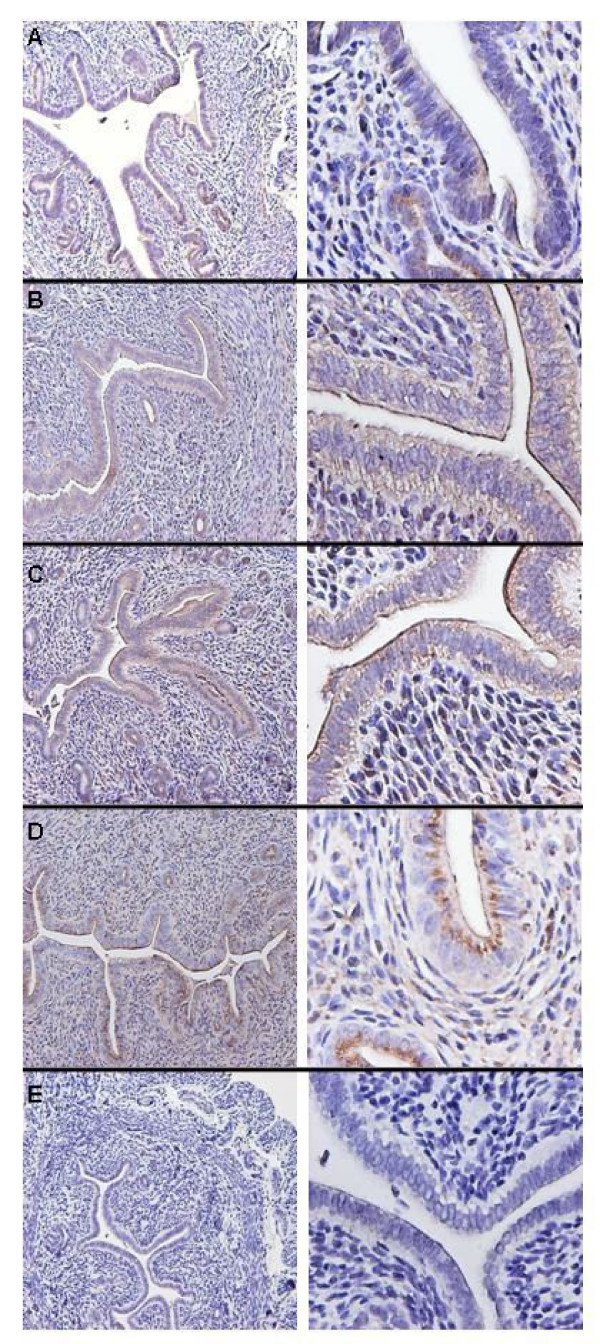
Progesterone induces surface expression of EMP2 in mouse endometrium in vivo. Mice were injected with progesterone or sesame oil (control) daily for up to 5 days. Tissue was harvested at days 1, 2, and 4 of treatment or 3 days following completion of treatment, processed for immunohistochemistry, and then stained for EMP2 expression (A-D) or a vehicle control (E). (A) 24 hours of progesterone treatment; (B) 48 hours of treatment; (C) 4 days of treatment; (D) 3 days following treatment; (E) representative control treatment at 4 days. Magnification: (left) 100×; (right) 400×. The experiment was repeated at least 3 times with similar results; representative images are shown.

At baseline (3–5 weeks after ovariectomy), EMP2 was not detected in the endometrium (data not shown). Within 24 h of progesterone injection, EMP2 was clearly detected in the glandular epithelium, but was only faintly detected in luminal epithelium at this time point (Fig. 5A). Within 48 h of progesterone injection, EMP2 expression on the apical plasma membrane of the luminal and glandular epithelium increased significantly (Fig. 5B); this increase persisted through day 4 of treatment (Fig. 5C). However, three days following completion of progesterone treatment (day 7), EMP2 protein was no longer detected at the apical plasma membrane, but was still observed in cytoplasmic compartments of both luminal and glandular epithelium (Fig. 5D). These changes in EMP2 expression were specific to progesterone as evidenced by the absence of an effect after injection solely of the vehicle control (Fig. 5E).

**Figure 6:**
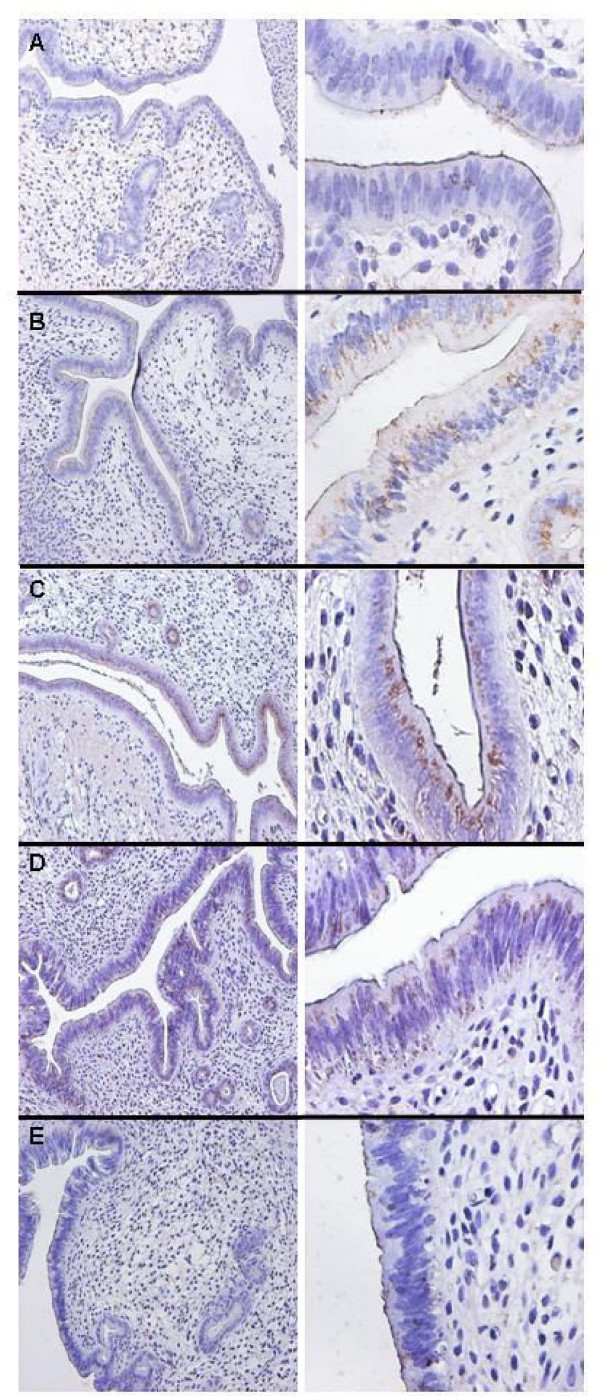
Estradiol induces cytoplasmic expression of EMP2. Mice were injected daily with estradiol for up to 5 days. Tissue was harvested at days 1, 2, and 4 of treatment or 3 days following estradiol treatment, processed for immunohistochemistry and stained with EMP2 antisera (A-D) or preimmune serum (E). (A) 24 hours of estradiol treatment; (B) 48 hours of estradiol treatment; (C) 4 days of estradiol treatment; (D) 3 days following estradiol treatment. (E) Representative isotype control at 48 hours of estradiol treatment. 100× (left side) and 400× (right side) images are provided. The experiment was repeated 3 times, and representative images are shown.

Treatment of ovariectomized mice with estradiol only modestly affected EMP2 expression in native endometrium (Fig. 6). No EMP2 expression was observed within 24 h of estradiol treatment (Fig. 6A). However, daily injection of 100 ng estradiol induced faint EMP2 expression by 48 h after initiating treatment (Fig. 6B). EMP2 expression was more prominent by day 4 of treatment (Fig. 6C), but was no longer observed 3 days following completion of treatment (Fig. 6D). No staining was observed with rabbit isotype control as the primary antibody (Fig. 6E). In contrast to progesterone, estradiol induced EMP2 expression only within the cytoplasmic compartment and not at the apical plasma membrane. That is, estradiol did not induce translocation of EMP2 to the plasma membrane surface during this experimental window.

## Discussion

Previous studies in a number of cell types suggest that EMP2 functions as a trafficking molecule, promoting the surface expression of cohort proteins such as integrins, GPI-anchored proteins, and MHC class I molecules [1,3-5]. In the endometrium, EMP2 expression modulates αvβ3 integrin expression, and EMP2 is required for successful implantation [1,3]. Moreover, dysregulation of EMP2 has been linked to endometrial adenocarcinoma, where upregulation of EMP2 is a prognostic indicator of advanced or high grade disease [2]. Because the estrous/menstrual cycle appears to regulate EMP2 expression in both mouse and human [1,2], we examined the role of steroid hormones in EMP2 regulation. In this report, we investigate the regulation of EMP2 using a combination of archived human tissue, the human endometrial cancer cell line RL95-2 *in vitro*, and hormonally manipulated native mouse endometrium *in vivo*. Our findings with these three biologic systems were consistent with the conclusion that progesterone significantly influences the protein expression and surface localization of EMP2.

In native human endometrial specimens, EMP2 protein was highly expressed in secretory human endometrium, at levels 3-fold higher than in proliferative endometrium. This secretory phase response suggested a role for progesterone and/or estradiol, and their relative contributions were assessed using the human RL95-2 cell line *in vitro*. In these cells, EMP2 protein was increased 2.5- to 4-fold after hormone treatment (72 hours). This response was dependent on progesterone, inhibitable by progesterone antagonist, and neither replicated by estradiol alone nor augmented in combination with estradiol. The same hormonal features (progesterone dependence, inhibition by progesterone antagonist, and minimal estradiol effect) were observed by immunohistochemical analysis of EMP2 protein expression in native endometrium of ovariectomized mice.

Mechanistically, this hormonal response did not appear to particularly involve transcriptional control, since EMP2 mRNA expression in RL95-2 cells was only modestly upregulated by progesterone or estradiol, reaching a peak at 4 hours after induction (~1.5-fold of vehicle control). These protein and mRNA results suggest that the EMP2 expression was predominantly controlled by progesterone rather than estradiol, and probably largely through a post-transcriptional effect of progesterone signaling on cellular processes that secondarily affected EMP2 protein levels.

In native mouse endometrium, previous studies have shown that EMP2 protein is expressed in a cytoplasmic pattern early in estrous/menstrual cycle, and on the plasma membrane during the window of implantation [1,2]. These findings had suggested that in addition to expression levels, hormonal induction of the endometrium might also elicit EMP2 translocation. This prediction was confirmed in the present study by laser confocal and conventional immunohistochemistry. Hormone treatment of RL95-2 cells induced a shift of EMP2 from a predominantly cytoplasmic to surface localization, in a process dependent on progesterone, inhibitable by progesterone antagonist, and unaffected by estradiol. Similarly, hormone treatment of native endometrium of ovariectomized mice *in vivo *also induced dramatic surface localization of EMP2, whereas estradiol had no effect on EMP2 surface translocation. The progesterone-induced translocation began at day 2 after treatment, suggesting that progesterone activates a molecular trafficking process required for efficient delivery of EMP2 to the cell surface. While the ligated progesterone receptor elicits profound changes in the cellular transcription program [16-19], we are not aware of studies that have delineated within this program a progesterone-induced membrane translocation system.

During implantation, hormonal and local signals instruct endometrial cells to acquire receptivity during the brief implantation window. Given the importance of EMP2 in regulating αvβ3 integrin, GPI-APs, and MHC I, the regulation of EMP2 is important to understanding the complex changes that occur during the implantation window. If EMP2 expression is an important physiologic switch for implantation competence, this implies that infertility in some cases may be due to genetic or biologic impairments in the program of endometrial EMP2 expression. Accordingly, agents that might overcome impaired EMP2 expression in the uterus could be therapeutically useful. Additional experiments will be important to validate the role of EMP2 in the implantation window.

## Abbreviations

4-TM: Four-transmembrane, tetraspan proteins; EMP2: Epithelial membrane protein-2; PMP22: Peripheral membrane protein-22; pc: Postcoital; MHC: Major histocompatibility complex; GPI: Glycosylphosphatidyl inositol.

## Authors' contributions

MW carried out immunohistochemistry and immunoflorescence experiments, and drafted the manuscipted. MM conducted in the in vivo hormone treatment experiments. SAM carried out the q-PCR studies. RGR performed the in vitro hormone treatment experiments. LKG participated in the design of the study. CJW and JB participated in the design of the study and helped to draft the manuscript. All authors read and approved the final manuscript.
